# Using AI in Optimizing Oral and Dental Diagnoses—A Narrative Review

**DOI:** 10.3390/diagnostics14242804

**Published:** 2024-12-13

**Authors:** Amelia Surdu, Dana Gabriela Budala, Ionut Luchian, Liliana Georgeta Foia, Gina Eosefina Botnariu, Monica Mihaela Scutariu

**Affiliations:** 1 Department of Oral Diagnosis, Faculty of Dental Medicine, “Grigore T. Popa” University of Medicine and Pharmacy, 700115 Iasi, Romania; 2 Department of Dentures, Faculty of Dental Medicine, “Grigore T. Popa” University of Medicine and Pharmacy, 700115 Iasi, Romania; 3 Department of Periodontology, Faculty of Dental Medicine, “Grigore T. Popa” University of Medicine and Pharmacy, 700115 Iasi, Romania; 4 Department of Biochemistry, Faculty of Dental Medicine, “Grigore T. Popa” University of Medicine and Pharmacy, 16 Universitătii Street, 700115 Iasi, Romania; 5 St. Spiridon Emergency County Hospital, 700111 Iasi, Romania; 6 Department of Internal Medicine II, Faculty of Medicine, “Grigore T. Popa” University of Medicine and Pharmacy, 16 Universitătii Street, 700115 Iasi, Romania; 7 Department of Diabetes, Nutrition and Metabolic Diseases, St. Spiridon Emergency County Hospital, 700111 Iasi, Romania

**Keywords:** artificial intelligence (AI), oral diagnosis, dental diagnosis, oral cancer

## Abstract

Artificial intelligence (AI) is revolutionizing the field of oral and dental healthcare by offering innovative tools and techniques for optimizing diagnosis, treatment planning, and patient management. This narrative review explores the current applications of AI in dentistry, focusing on its role in enhancing diagnostic accuracy and efficiency. AI technologies, such as machine learning, deep learning, and computer vision, are increasingly being integrated into dental practice to analyze clinical images, identify pathological conditions, and predict disease progression. By utilizing AI algorithms, dental professionals can detect issues like caries, periodontal disease and oral cancer at an earlier stage, thus improving patient outcomes.

## 1. Introduction

The broad definition of artificial intelligence (AI) is the ability of machines and other technological systems to mimic human intelligence and carry out previously human-only tasks [1]. These tasks include problem-solving, decision-making, learning, and understanding natural language. The concept of AI has evolved significantly since its inception in the mid-20th century, moving from theoretical frameworks to practical applications across various industries [1,2]. Over the last decade, artificial intelligence (AI) has attracted a great deal of attention, with healthcare quickly surpassing all other AI applications in terms of overall equity investment [2]. At its core, AI relies on algorithms and computational models to process large datasets, identify patterns, and make predictions or decisions based on those patterns [3]. These algorithms, often rooted in machine learning and deep learning techniques, enable systems to improve their performance over time as they are exposed to more data [4].

By automating the analysis of complex datasets, AI can uncover insights that may be difficult or impossible for humans to detect. This capability allows for enhanced accuracy and efficiency across diverse applications, ranging from predictive analytics to real-time decision-making in dynamic environments [2,3].

Artificial intelligence (AI) is well suited to the diagnostic imaging industry because it can reduce costs by doing away with routine operations, improve the efficacy of treatment, and eliminate the inherent heterogeneity in subjective individual examinations. 

Already, AI is having a profound effect on several industries, including healthcare, banking and transportation, and this influence is only going to increase. Intelligent tutoring systems, computer programs that can adapt to the demands of individual pupils, have been developed in academia using AI [2]. Students’ performance in math and science, among other topics, has been enhanced by these methods. Advancements in areas like genetics and medication development have resulted from the application of AI to study massive datasets and spot patterns that people might miss. AI has already made great strides in the medical field, allowing for more precise diagnoses and more tailored treatment programs. Responsible and inclusive AI development is of the utmost importance as technology advances [2,3].

With AI developing at such a rapid pace, there is a chance that it may change healthcare as we know it by being applied in clinical practice. In order to ensure that healthcare practitioners have the information they need to effectively incorporate AI into patient care, it is crucial to record and share data about AI’s function in clinical practice.

Just like in other professions, AI is undergoing a transformation and is now being used in dentistry. From helping with clinical diagnosis and treatment planning to scheduling and managing regular appointments, AI can do many simple jobs in the dental clinic more accurately, with fewer workers, and with fewer mistakes than humans [3,4].

Essentially, the field of artificial intelligence has witnessed a meteoric rise in technological advancements over the past decade. Nevertheless, it remains unclear from the existing literature how AI-related data might aid in the detection, prevention, and treatment of dental problems [4]. We conducted a review that addressed various modalities of artificial intelligence, its applications and outcomes in dentistry to gain a better understanding of the current trends of AI in this field, especially in oral and dental diagnoses.

The purpose of this narrative review was to present a synopsis of the literature on the topic of AI’s role in improving dentistry and oral health diagnostics. The literature search was conducted with rigor and transparency in mind using a structured, non-systematic method:

✓ Databases and search strategy: the search was performed across PubMed, Scopus and Web of Science. Keywords which were used in the search included “artificial intelligence”, “oral diagnosis”, “dental diagnosis”, “machine learning”, “deep learning”, and “AI applications in dentistry.” The search results were refined using Boolean operators (such as AND and OR).
✓ Inclusion criteria: Peer-reviewed studies addressing the application of AI in oral and dental diagnoses, including diagnostic imaging, disease detection, and AI-based tools.
✓ Exclusion criteria: articles focusing on non-diagnostic aspects of dentistry, AI tools outside the scope of oral and dental health.
✓ We prioritized studies published in the last 10 years to ensure that the review captures recent advancements, but seminal works outside this range were also included if they contributed significantly to foundational knowledge.
✓ Selection criteria: Titles and abstracts were screened for relevance, followed by a full-text review of selected articles. Emphasis was placed on including studies that provide insights into both the benefits and limitations of AI in oral and dental diagnoses. The articles were categorized into themes based on their focus areas (e.g., diagnostic imaging, disease detection, AI-based treatment planning) to structure the discussion logically

## 2. Exploring the Applications of AI in Dentistry

Quickly transitioning from a theoretical notion to a practical tool utilized in sectors like healthcare, banking, and manufacturing, artificial intelligence (AI) has come a long way. Artificial intelligence (AI) is the study and development of computer programs with the ability to learn and solve problems in a way that is comparable to human intelligence [5]. Numerous systems have emerged as a result of AI’s expansion; these systems can be categorized according to their capacities and methods of learning.

Artificial intelligence (AI) plays a pivotal role in modern dentistry by enhancing diagnostic precision, optimizing treatment planning, and improving overall patient care. Through advanced algorithms and machine learning, AI systems can analyze complex data, such as radiographs and patient records, to identify patterns and support clinical decision-making. These technologies help reduce human error, increase efficiency, and streamline workflows in dental practices. Additionally, AI enables personalized treatment approaches by tailoring recommendations to individual patient needs. Its integration into areas such as orthodontics, endodontics, and oral pathology highlights its versatility and potential to transform dental care. As AI continues to evolve, its applications in dentistry are expected to expand further, improving accessibility and outcomes.

**Figure 1:**
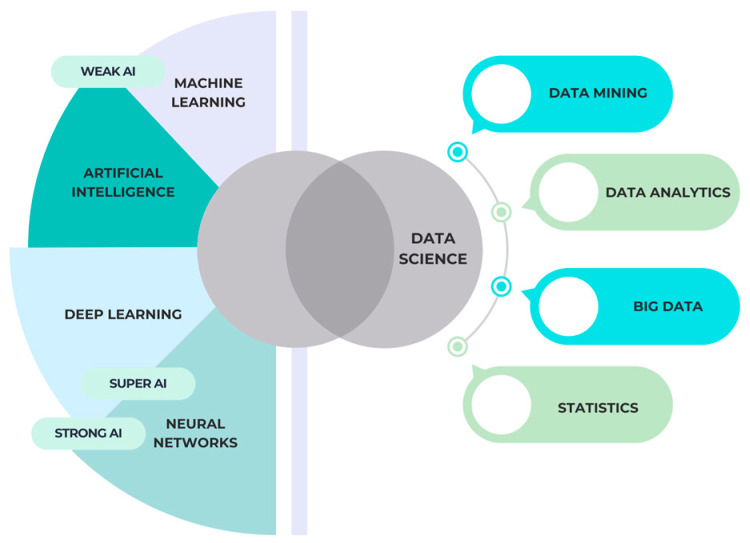
Artificial intelligence (AI) forms and their interaction with data science.

There are three distinct phases of artificial intelligence: weak, general, and super, or genius. Today, the majority of AI applications, including data mining, facial recognition, and reinforcement learning, fall under the category of narrow AI, which is created to address certain tasks. Strong AI strives to be as smart and self-aware as humans and capable of making judgements in a variety of domains. Achieving an intelligence level higher than humans is the ultimate aim of super or genius AI. The three forms of artificial intelligence, their respective domains, and how they interact with data science are illustrated in Figure 1.

Systems that employ artificial intelligence rely on data and algorithms to function. Mathematical models, sometimes known as algorithms, are trained using large amounts of data in order to identify trends and make predictions. Algorithms are put into use in different applications after training, and they are constantly updated and improved by fresh data. This paves the way for AI systems to gradually become more efficient and accurate in performing complicated tasks such as data analysis, language processing, and identifying images. 

**Figure 2:**
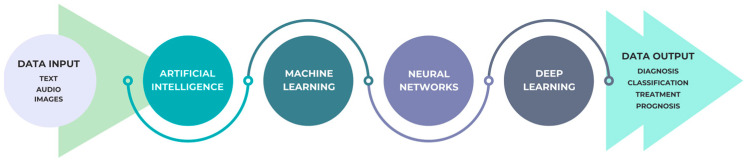
A simplified model of AI’s operation.

Neural networks (NNs), machine learning (ML), and deep learning (DL) are the fundamental building elements of contemporary artificial intelligence (AI) and are crucial to its progress, and Figure 2 captures the typical workflow of an AI solution.

In a mathematical nonlinear model, neural networks (NNs) recreate the human brain by using artificial neurons that are analogous to human neural networks. Problem-solving, learning, and decision-making are all examples of human cognitive abilities that NNs may mimic [6]. Typology, network structure, and learning algorithm are the three main ways to categorize neural networks (NNs). One way to identify a network is by looking at its topology, which may tell us things like how many layers it has, whether those levels are visible or hidden, whether they are input or output, and which way the connections between neurons point. We can differentiate between monolayer and multilayer networks based on their typology [7,8]. NNs can detect tooth cavities, identify anatomical features, and supplant cone-beam computer tomography (CBCT), which uses high radiation doses, in the endodontics area. Oral pathology also relies on them heavily [9,10]. Numerous neural network types are available for use in various contexts.

Layered neural networks enable deep learning, a kind of machine learning, to model complicated patterns in massive datasets. Its ability to simulate neural processing has led to the development of AI systems with remarkable capabilities in areas such as picture identification and natural language processing (NLP) [9]. A network of interconnected “neurons” (computer components) that mimic the way the human brain processes data and learns new tasks is called a neural network [10].

One branch of artificial intelligence, machine learning, allows computers to learn from their own data and get better over time without human intervention. A machine learning algorithm uses statistical techniques to help it learn how to get progressively better at a task, without necessarily having been programmed for that certain task. It uses historical data as input to predict new output values. According to Goodfellow et al. [11], ML algorithms have the ability to recognize patterns, make judgements, and forecast events using the data that are inputted. Convolutional neural networks (CNN) are another name for deep learning (DL). For the most part, they handle complicated and substantial images by collecting features from the abstracted filter layer.

One way to describe deep learning (DL) is as ML on steroids [12]. Among the many branches of machine learning, deep learning stands out. It makes use of a specific kind of artificial neural network called a deep neural network. These networks have many hidden layers that process data in a specific way, enabling machines to learn more complex patterns, establish connections, and optimize input by weighting it [12].

To implement this complex type of machine learning, programmers have turned to artificial neural networks (ANN), which are algorithms designed to mimic the way the human brain operates. A deep learning artificial neural network can include anything from a few thousand to a few million neurons distributed across three layers—the input, the output, and the hidden layers—in contrast to the human brain’s about 85 billion synaptic connections.

Pathologies affecting the teeth and oral cavity rank high among the most common medical issues affecting humans. There is a shortage of dental and oral health care professionals, which is adding strain to already overburdened health care systems and jeopardizing the affordability and accessibility of dental and oral health care in the face of anticipated increases in the burden of these diseases caused by demographic and epidemiological trends [13]. Many people believe that digital technologies, such artificial intelligence (AI), can improve decision-making and process efficiency. In the field of oral and dental care, AI holds immense promise for improving the quality of treatment for a wider range of patients through increased efficiency, safety, and efficacy [14].

Neural networks, machine learning, and deep learning work together to create AI systems that can learn and get better over time, which opens up a world of possibilities for technological advancement [15,16]. The complete use of AI for medical diagnostics is still in its early stages. On the other hand, more information is coming out on the use of AI to diagnose various oral disorders.

Due to technological improvements and the digitization of dentistry, artificial intelligence is expanding in the field of dentistry. In several dental specialties, computers may now provide second perspectives. The application of AI in dentistry has the potential to greatly improve the speed, accuracy, and efficiency of diagnosis. 

## 3. AI Applications in Diagnosing Oral Conditions

► Caries Detection

One of the most frequent long-term health problems is dental caries, or tooth decay [17]. The present school of thought in caries management follows a medical paradigm that emphasizes preventing caries, managing caries without restoration, and treating caries with less invasive surgical procedures [18]. Preserving healthy dental hard tissue through early intervention is made possible by identifying dental caries, particularly at an early stage. Consequently, in order to prevent and manage dental caries, it is crucial to diagnose and assess the condition, particularly in its early stages [19,20]. 

There is a need for new methods of detecting caries because the old ones have their limits. A number of new technologies have been created, one of which is a computerized diagnostic tool that may detect lesions in their early stages. Various sectors have seen a surge in the use of digital aids, which are gadgets that use digital data for caries diagnostics.

Deep learning with convolutional neural networks (CNNs) has been the subject of much investigation in the field of medical image processing in recent years, and early results show encouraging results. A growing number of medical professionals are turning to deep learning for illness diagnosis because it improves clinical outcomes through rapid, accurate identification [20].

Using AI technology, Hung et al. [21] were able to successfully predict root caries. Also, in panoramic dental radiographs, Ekert et al. showed that CNNs successfully identify apical lesions (ALs) [22]. When compared to dentists with 3–14 years of expertise, a CNN model performed better in Cantu et al.’s study of first carious lesions [23]. Similarly, while utilizing bitewing radiographs to diagnose proximal caries, Devito et al. [24] discovered encouraging results using an ANN model.

In particular, these models still struggle when it comes to detecting cavities in molars and other teeth with intricate morphology. One research found that convolutional neural network (CNN) models performed better in identifying caries in premolars than in molars, which have a more complicated morphology [25]. In most instances, these AI models achieved accuracy levels above 92%, indicating good overall performance. As an example, compared to conventional approaches, Geetha et al. found that ANNs could diagnose caries with a 97.1% accuracy rate [26]. In contrast, Dayi et al. discovered that a deep learning-based AI system performed poorly when it came to detecting cervical caries, even if it was good at detecting occlusal and proximal caries [27]. Using AI models on smartphone images, Duong et al. produced a prediction accuracy of 92.37% and a detection accuracy of 87.39% in another model [28,29]. A further drawback is that the majority of the existing research on automated caries identification is image-centric, ignoring patient history and clinical examination results that dentists normally take into account when making a diagnosis [30].

Recognizing and addressing risk factors that contribute to the advancement of caries has become more important with the trend towards preventative and minimally invasive dentistry. When used in conjunction with clinical evaluations, ML models may estimate the likelihood of root caries based on demographic and lifestyle factors. By sifting through massive information, these models reveal factors that could otherwise go unnoticed [31].

A 97.1% success rate, 95.1% precision rate, and 99.6% sensitivity rate were recorded by the AI-based ML model developed by Hung M et al. for the purpose of diagnosing root caries [27]. Similarly, Pang et al. [32] presented an artificial intelligence (AI) ML model that predicts caries risk based on environmental and genetic variables. However, this model has limitations due to the dataset’s variability.

When it comes to dental caries diagnosis and risk prediction, ML and DL are both seen to be useful approaches. Having access to appropriate and highly annotated datasets is crucial for the success of these applications [33].

► Pediatric Dentistry

Not only does the majority of the population not have a yearly oral exam, but oral health is often overlooked by people, compared to other sections of the body.

Finding that both human intuition and machine algorithms agreed on how the replies should be divided, Gajic et al. [34] analyzed the influence of dental health on adolescents’ quality of life using statistical approaches and artificial intelligence algorithms. In contrast to the more obvious categorization of respondents based on gender, artificial intelligence algorithms may be used to split the respondents into various categories. This enables the discovery of information that would not have been possible before.

Dental professionals, parents, and even children might utilize the findings from the machine learning toolbox to assess a patient’s dental health and determine if treatment is necessary. Dental schools must promote digital literacy in the next generation of dental professionals in order to support the integration of clinical AI technologies. Therefore, it is very advantageous to use machine learning in dentistry, as it allows us to obtain faster and more accurate outcomes [35,36].

One use of AI in mesiodens diagnosis is the use of individual deep learning models [37]. Screening results from young, inexperienced dental staff sometimes fail to reveal the existence of extra teeth on panoramic radiography [38]. Furthermore, there is a dearth of general dentists who are competent in the diagnosis of youngsters with mixed teeth. Despite these limitations, deep learning based on convolutional neural networks (CNNs) may offer substantial assistance in the screening of extra teeth [39]. 

The application of a deep learning model to identify mesiodens in primary or mixed dentition was demonstrated by Ahn, Y. et al. [40], suggesting that this approach might assist physicians with little clinical expertise in making more precise and prompt diagnosis. While two deep learning models outperformed human evaluators in terms of speed, they were only marginally more accurate than humans when it came to detection, which was far quicker. 

A fully automated method for identification was feasible, but the quantity and location could not be determined, according to Kim J. et al. [41], who used a deep learning system to diagnose mesiodens.

Kuwada et al. [42] found that two deep learning algorithms that can identify maxillary impacted supernumerary teeth on panoramic radiographs. Nevertheless, it should be mentioned that this identification is a challenge because the patient still has some permanent teeth that have not yet erupted. 

However, further development is necessary for clinical applications before the CNN-based deep learning approach can be utilized, despite its promise as a tool to aid dentists in their diagnostic work. Not long from now, it will be necessary to build a comprehensive diagnostic system that can handle a wider range of ages and diseases. Consequently, pediatric dentists may be able to establish treatment plans early with the use of CNN-based deep learning, which might enhance screening by non-pediatric dentists.

► Orthodontics

Orthodontics is a branch of dentistry that focusses on the diagnosis of malocclusion and the development of systematic treatment plans. In most cases, malocclusion hinders occlusal function, which in turn causes psychological pain and, in the long run, decreases the quality of life [43,44].

The most important tool for orthodontic diagnosis is cephalometric radiographs, which are taken to evaluate any anomalies related to the craniofacial skeleton. Other important components of an orthodontic diagnosis include the patient’s medical and dental history, a clinical examination, study models, and the patient’s occlusion [45].

Results from an investigation on the efficacy of cellular neural network-based models for accurately localizing cephalometric landmarks on cephalometric radiographs were promising. The study’s encouraging results and the model’s performance being comparable to the specialists were similar to those of another study that used paraconsistent artificial neural networks (PANN) to analyze the cephalometric variables for orthodontic diagnosis [46].

In a separate study, convolutional neural networks (CNNs) were employed for automated quantitative cephalometry in order to identify cephalometric landmarks. The system outperformed the top benchmarks reported in the literature with an accuracy rate of 76% [47]. These findings are in line with those of a previous study that also employed CNN-based models, which achieved results comparable to those of human experts. The results were encouraging [48,49,50].

An artificial intelligence system developed by Kunz et al. was evaluated in their study as an accurate method for automatically tracing cephalometric landmarks [51]. The results showed that the “gold standard” method of manually localizing locations had a very high correlation with AI predictions.

With the goal of automatically identifying sites on cephalometric radiographs, Kim et al. developed their own AI-powered algorithm [52]. The study’s findings suggest that deep learning models can outperform experienced physicians for some landmarks; evaluating the efficacy of AI detection of cephalometric landmarks relies heavily on inter-examiner variability.

The initial study to assess the efficacy of an automated method for the identification of cephalometric landmarks in individuals with both whole and partial permanent teeth was conducted by Tanikawa et al. [53]. The authors came to the conclusion that the system was able to identify all the surrounding anatomical features of all the markers.

The majority of the prominent AI algorithms can analyze a cephalometric radiograph in a matter of seconds, according to the studies. Compared to physicians manually tracing landmarks, this takes a lot less time. Modern algorithms are becoming more efficient and dependable as a result of their fast evolution and greater computing capability [54,55,56,57].

In the future, AI systems that use automatic landmark localization might be more effective than human tracers. Although AI-generated interpretations of cephalometric data may not always match the level of expertise exhibited by human orthodontists, they can nonetheless prove useful for less seasoned practitioners or individuals without specific education [58,59]. When it comes to orthodontic treatment planning, monitoring, and analysis, further study is needed to establish the correctness of cephalometric analyses performed by AI. Patients may benefit greatly from an improvement in the efficiency of their orthodontic treatment if artificial intelligence enables quick and painless cephalometric examination [60,61].

► Periodontal Disease

The inflammatory reaction of the human immune system to various bacterial species in the mouth cavity causes periodontal disease, a complex disorder with several causes [62]. By detecting periodontal disease early on with AI, patients can have better oral health, which in turn improves their general well-being and quality of life [63].

In order to diagnose and treat periodontitis, it is essential to recognize periodontal bone loss early on, which might be challenging for inexperienced practitioners [64]. Danks et al. [65] evaluated periodontal bone loss and its severity with a total accuracy of 83.3%, using a deep neural network on periapical radiographs from 63 individuals.

Periapical radiolucency and alveolar bone resorption are two characteristics that may be used to train AI models to detect periodontitis and other diseases in the gums and surrounding tissues. Discovering periodontally compromised premolars and molars and forecasting hopeless premolars and molars according to the degree of alveolar bone loss is made possible with a model, provided by Lee et al., based on deep learning convolutional neural networks [66].

A deep learning model was also employed to stage periodontitis by detecting and quantifying periodontal bone loss from panoramic pictures. Three oral radiologists’ results were compared to the deep learning models. Automatic periodontal disease diagnosis and routine surveillance might both benefit from the DL model’s high accuracy and reliability [67].

Additionally, ANN-based tests were performed in periodontology to identify chronic periodontitis (CP) and aggressive periodontitis (AgP) caused by autoimmune disorders. When asked to categorize patients into one of the categories, the ANNs achieved an accuracy rate of 90–98% [68]. Applying ANN with cross-entropy (CE) monocytes, eosinophils, neutrophils, and CD4/CD8 as inputs yielded the best overall predictor. Consequently, ANNs may reliably diagnose AgP or CP using easily available and relatively basic criteria, including the peripheral blood leukocyte count. Because of this, practitioners will be able to provide more personalized treatment plans for their AgP and CP patients [68].

► Oral Malignancies

Similar to other medical fields, oral and maxillofacial (OMF) pathology raises a number of ethical and regulatory concerns about the use of AI. Data from patients are crucial to the operation of artificial intelligence systems employed in OMF pathology, especially those that process diagnostic images [69,70,71,72]. The EU’s General Data Protection Regulation (GDPR) is one example of privacy legislation that must be followed while collecting, storing, and using this type of data [73]. Data security and the prevention of unauthorized access also necessitate rigorous safeguards. The use of artificial intelligence (AI) in healthcare has both positive and negative aspects that patients should be aware of. When artificial intelligence models are employed for diagnostic reasons, informed consent takes on further significance [74,75]. 

Any biases present in the datasets used to train AI systems run the risk of being perpetuated by the AI itself. A common limitation of AI systems is that they are often incomprehensible to doctors because of their black box nature. In a clinical context, when knowing the logic behind a prognosis or diagnosis is vital, this might be a problem. Thorough testing is necessary to guarantee the safety and efficacy of AI systems utilized in OMF pathology. Additional randomized control trials are required to confirm the findings of studies like Kuwada et al. [76] and Al Sarem et al. [77], which give useful data. For these AI systems to be validated and approved, regulatory agencies need to establish criteria.

Physicians have difficulty when it comes to accurately diagnosing and classifying different maxillofacial cysts and tumors. There is promising future clinical use of AI for the automated diagnosis of certain disorders. To better categorize maxillofacial lesions and tumors, researchers are concentrating on building artificial intelligence models that are taught using 2D/3D pictures [78,79]. 

A completely automated model that can detect cysts and tumors is still a challenge to construct, and manual input is still required for the early phase of lesion identification. Notably, Santer et al. discovered that AI shows promise in identifying suspicious lymph nodes in patients with locally advanced head and neck squamous cell carcinoma in a comprehensive analysis that included thirteen researchers. In their study, the authors proved that AI could detect lymph nodes with an average accuracy of 86% [80].

A major concern in public health, oral cancer is among the most common cancers and has a high fatality rate. Since AI requires complicated data on etiology and risk variables, the most difficult parts are oral cancer diagnosis and recurrence prediction [81,82,83]. When compared to other methods for oral cancer detection, AI offers clear advantages. Because of its adaptability, this innovation can always teach more. With the addition of fresh patient data, AI computations can enhance their predictive capabilities, which in turn lowers treatment costs and alleviates patients’ financial burdens [83]. The rapid and accurate diagnosis of OC is possible with the application of AI learning systems trained on data collected from cytology, fluorescence, CT, and depth of invasion images.

Tele cytology (TC), the digitization of cytology slides, was used in a piece of research by Sunny et al. [84] to identify OC early on. An enhanced accuracy of 93% in detecting malignancies and 73% in identifying possibly malignant lesions were demonstrated by the ANN-based model.

In their study, Jeyaraj et al. [85] employed a regression-based deep-learning approach to detect OC and describe the progression of oral cancer. They discovered that the regression-based method was 91.4% sensitive in detecting malignant lesions when tested on identical images as the conventional methodology. The proposed algorithm model outperformed the gold standard in terms of diagnostic accuracy.

Using convolutional neural networks (CNNs), Uthoff et al. [86] were able to distinguish between precancerous and malignant lesions in white light and autofluorescence photos. When compared to experts, CNN was more accurate in diagnosing malignant and precancerous tumors.

In order to diagnose cancer, AI algorithms may sift through several data sources, evaluate risk, and recommend experts. Research on lymph nodes, premalignant lesions, and cancerous tumors has revealed promising findings for the diagnostic and prognostic use of AI. If these initiatives are successful in facilitating early diagnosis and curative treatments, they may lower death rates.

► Maxillary Sinus Condition

Extraoral radiographs are a typical tool for visualizing structures such as the maxillary sinuses. Misdiagnoses may be drastically reduced with the use of AI that can automatically identify sinuses and detect any pathology within them. For the purpose of diagnosing maxillary sinusitis utilizing panoramic radiographs, Murata et al. [87] tested a DL system. Two radiologists and two residents were used to compare the AI’s performance. In terms of diagnosis accuracy, the system was on par with the radiologists. Having said that, AI outperformed the dental residents [87]. 

Using maxillary sinus radiographs taken from Water’s perspective, Kim et al. investigated how well the DL system could diagnose problems. AI greatly improved the sensitivity and specificity of radiologists, as shown in research [88].

► Salivary Gland Diseases

Because of their similar and often misleading appearance, salivary gland illnesses can be difficult for novice dentists to diagnose. In the context of salivary gland disorders, AI has the potential to be an invaluable diagnostic tool. DL models have the potential to outperform radiologists in some cases [89]. The low incidence and common morphologic features of salivary gland tumors make them difficult to detect. Employing ML allowed for the identification of malignant salivary gland tumors based on their cytologic appearance. Comparing its findings to those of trained medical experts was part of the evaluation process. The accuracy of pathological condition identification was enhanced by the decision tree system test, which reduced the number of potential diagnoses [90].

Among the many sectors being transformed by AI is the dental industry. The adoption of AI has already started improving certain patient care and reducing operations, similar to contemporary dentistry technology. 

In Table 1 below, we look at artificial intelligence (AI) in dentistry and the cutting-edge ways it is changing the face of dentistry today. 

In order to offer an accurate and economical diagnosis, these platforms will need massive datasets and the means to analyze them. We need to enhance these models to achieve the best accuracy with specificity and sensitivity so they may be securely integrated into daily clinical processes.

## 4. Future Perspectives

The area of artificial intelligence (AI) has recently gained momentum in the scientific and technical communities. Image processing is fundamental to artificial intelligence, which in turn is vital to the field of dentistry. Continuous patient assessment and monitoring, pharmacological long-term effects, and health-related risk prediction are all areas where AI has the potential to have a significant impact [91,92].

Artificial intelligence (AI) might one day make dentists’ lengthy hours obsolete. It is also possible to integrate healthcare for all, provide personalized, preventive, and predictive dental treatment, and enhance people’s health while reducing expenses. The most important ways in which AI could improve dental care are as follows: by making diagnoses more precise and effective; by creating more realistic treatment visualizations; by simulating treatment outcomes and predicting oral health and illness [93,94,95,96]. 

As supplementary tools, AI models have also attracted interest for improving the accuracy and precision of diagnosis. Artificial intelligence (AI) has several potential uses in healthcare and has already proven to be very effective in many areas, such as illness diagnosis and risk assessment [97,98,99]. Artificial intelligence in dentistry presents considerable potential to conform to and improve compliance with established diagnostic protocols. The American Dental Association (ADA) establishes extensive guidelines for the diagnosis of dental caries and periodontal disease.

AI models developed for caries detection utilize these principles to guarantee that their diagnostic procedures are clinically pertinent and precise. Likewise, the World Health Organization (WHO) oral health standards underscore the significance of early detection and standardized diagnostic processes, both of which are enhanced by AI’s capacity to analyze extensive datasets and recognize first pathogenic alterations [100].

The European Federation of Periodontology (EFP) recommendations emphasize the necessity for the precise identification of periodontal bone loss and severity evaluation in the diagnosis of periodontal disorders. AI-driven systems, including machine learning models, closely adhere to these requirements by providing accurate measurements and automated assessments, hence minimizing diagnostic variability [101].

By integrating AI technologies informed by these diagnostic frameworks, dental practitioners may attain enhanced accuracy, consistency, and efficiency, thereby enhancing patient outcomes and optimizing compliance with international standards. This collaboration highlights the revolutionary impact of AI in updating diagnostic techniques while following the conventional guidelines [102].

Numerous chances and difficulties lie ahead in the future. Innovation and new technology are expected to propel further progress, changing the way industries function and enhancing results in several fields. To meet changing demands and make sure future solutions are long-lasting, research and development must be ongoing. Furthermore, new opportunities for development and advancement will present themselves in the future as a result of the incorporation of new tools and approaches [103].

AI systems must also account for diverse patient populations, ensuring models are trained on representative datasets to reduce disparities in outcomes. Additionally, regulatory, educational, and infrastructural differences between countries impact the pace and scale of adoption, requiring tailored approaches to meet local needs. Technological challenges include biases in training datasets and the need for more robust models to handle complex and atypical cases accurately. System accessibility remains a significant issue, especially in low-resource settings where the lack of infrastructure and expertise hinders implementation. By addressing these challenges, the dental field can fully harness AI’s potential to improve diagnostic accuracy, optimize treatment planning, and enhance patient care on a global scale.

The broad application of image analysis driven by AI is one of the major advances that are expected to happen soon. Artificial intelligence algorithms can now identify oral cancer, periodontal disease, and dental caries in their early stages with an accuracy that often exceeds that of humans [104]. More sophisticated algorithms analyzing massive amounts of information from 3D imaging, CT scans, and radiography will likely enhance these capabilities in future systems, allowing for more accurate, real-time diagnosis. More proactive and preventive treatment is possible with the use of these technologies, which will enhance early identification and aid in illness progression prediction [105].

Improvements in natural language processing (NLP) will also allow AI systems and physicians to communicate more smoothly, which will aid in the automation of patient records, the simplification of diagnostic processes, and the improvement in decision-making based on data-driven insights.

New digital diagnostic tools and their clinical usage are the main topics of this study. Also noteworthy are the methods that have been created to improve the precision with which computerized diagnostic aid data may be understood. These systems should be seriously considered for use in clinical practice based on the findings of the research that have been reported. For less experienced dentists, these devices can be an excellent supplementary assistance.

## 5. Limitations of the Review

There should be an acknowledgement of the review’s limitations despite the fact that it offers a thorough summary of the numerous digital diagnostic tools now used in clinical practice for the diagnosis of oral disorders.

There is a lack of clinical evidence about the diagnostic accuracy and possible influence on clinical application, such as patient experience and satisfaction, for many digital diagnostic aids that are now accessible in clinical settings. This is especially true because many aids are relatively new. The potential monetary and logistical costs and benefits of using these diagnostic tools were also not thoroughly addressed. It is highly expected that in the future, further research may be carried out to create and evaluate AI diagnostic systems that are both efficient and effective.

## 6. Conclusions

Dental and oral healthcare are only two of many areas that have seen a dramatic improvement in diagnostic skills because of the advent of AI in healthcare. Professionals have traditionally depended significantly on manual evaluations to diagnose oral disorders such dental caries, periodontal problems, and oral malignancies. While this method is successful, it might be constrained by clinicians’ subjective interpretations and unpredictability. The use of AI in healthcare has greatly improved the precision of diagnosis and treatment planning. When put to use as intended, these technologies have shown to be remarkably effective.

Although artificial intelligence (AI) provides considerable improvements in diagnostic precision, efficiency, and early identification, it should not operate as an independent instrument for detecting complex diseases, such as Alzheimer’s disease. AI systems are proficient in analyzing extensive datasets, recognizing complex patterns, and minimizing human error, but they are deficient in the contextual comprehension and critical reasoning that human physicians contribute to the diagnosis process.

Artificial intelligence is most effective when utilized as an assistive tool, augmenting human knowledge by delivering data-driven insights to facilitate healthcare decision-making. Integrating AI’s computational capabilities with the nuanced judgement and ethical concerns of healthcare practitioners might result in more accurate, dependable, and patient-centered outcomes. The partnership between AI and human knowledge establishes a comprehensive diagnostic framework that utilizes the advantages of each, guaranteeing optimal treatment standards.

The papers included in this study provide evidence that AI-based systems have promising and dependable accuracy. Dentists may save time and work more efficiently with the aid of these technologies since they simplify chores and offer results quickly. Oral health diagnostics have become more accessible, efficient, and accurate with the use of artificial intelligence (AI), especially deep learning (DL) and machine learning (ML). 

Advanced data techniques for building unbiased datasets, generalized algorithm development, processing power optimization, and the establishment of clear ethical standards for responsible AI integration in dental healthcare are all crucial areas that need further investigation if we are to meet the present challenges head-on.

## Figures and Tables

**Table 1:** AI Applications and outcomes in diagnosing oral conditions.

Oral Condition	AI Application	Key Findings	Reference
Dental Caries	AI models for detection and diagnosis	High dental caries detection accuracy, early diagnosis and prevention.	Khandelwal, A. et al. [19]
Periodontal Diseases	AI in detecting periodontal bone loss	Effective identification of periodontal bone loss, aiding early intervention and management.	Danks, R.P. et al. [65]
Oral Cancer	AI for early detection and diagnosis	High diagnostic accuracy, facilitating timely treatment for better patient outcomes.	Khanagar, S.B. et al. [74]
Maxillary Sinus Diseases	AI in diagnosing sinus conditions	Improved accuracy in diagnosing sinus diseases, enhancing treatment planning.	Murata et al. [87]
Salivary Gland Diseases	AI in detecting salivary gland pathologies	Enhanced accuracy in diagnosing salivary gland diseases, contributing to better treatment outcomes.	López-Janeiro, Á. et al. [90]
Oral Disease Severity Assessment	AI for assessing the severity of oral diseases	High accuracy in evaluating the severity, aiding clinicians in personalized treatment planning.	Patil, S. et al. [63]
